# Sintering Quality Prediction Model Based on Semi-Supervised Dynamic Time Feature Extraction Framework

**DOI:** 10.3390/s22155861

**Published:** 2022-08-05

**Authors:** Yuxuan Li, Chunjie Yang, Youxian Sun

**Affiliations:** State Key Laboratory of Industrial Control Technology, College of Control Science and Engineering, Zhejiang University, Hangzhou 310027, China

**Keywords:** LSTM, semi-supervised learning, FeO content, soft sensor, encoder-decoder, dynamic feature extraction

## Abstract

In the sintering process, it is difficult to obtain the key quality variables in real time, so there is lack of real-time information to guide the production process. Furthermore, these labeled data are too few, resulting in poor performance of conventional soft sensor models. Therefore, a novel semi-supervised dynamic feature extraction framework (SS-DTFEE) based on sequence pre-training and fine-tuning is proposed in this paper. Firstly, based on the DTFEE model, the time features of the sequences are extended and extracted. Secondly, a novel weighted bidirectional LSTM unit (BiLSTM) is designed to extract the latent variables of original sequence data. Based on improved BiLSTM, an encoder-decoder model is designed as a pre-training model with unsupervised learning to obtain the hidden information in the process. Next, through model migration and fine-tuning strategy, the prediction performance of labeled datasets is improved. The proposed method is applied in the actual sintering process to estimate the FeO content, which shows a significant improvement of the prediction accuracy, compared to traditional methods.

## 1. Introduction

The iron and steel industry is the basic industry of the country, and it is also an energy-intensive process. The energy consumption and emissions of the steel industry account for a high proportion of a country’s industry. However, the current iron and steel industry is still at a low level of automation and informatization, and there are still many problems. For example, in the production process, it is difficult to obtain key information, establish process control models, and rely on manual experience. Therefore, for example, the product quality is unstable and the working condition is unstable.

The sintering process is the first key production process in the iron and steel industry. It provides raw materials for subsequent blast furnace ironmaking and determines the quality basis of subsequent processes. If the quality of iron ore sinter is poor and fluctuates greatly, the smelting process of blast furnace will be greatly affected, such as unstable working conditions and poor molten iron quality. Moreover, the sintering process is one of the most energy-intensive links in the iron-making process. The whole sintering process mainly depends on coal and coke as fuel, which produce a large amount of carbon emissions. To achieve the goals of improving quality, improving production efficiency, saving energy, protecting the environment, and sustainable development, the intelligent sintering process will become a research hotspot in academic and industrial circles in the future. Therefore, it is necessary to study the quality prediction of the sintering process. A prediction model of FeO is established to predict the quality in time, provide effective guidance information for operators, and control the production process timely and accurately. An accurate prediction model is helpful to improve the production quality and efficiency of the sintering process.

Generally speaking, industrial process modeling is divided into the mechanism model and data model [1]. The mechanism of some industrial processes based on physical processes is relatively simple, and the mechanism model is relatively easy to establish. When complex physical and chemical reactions are involved, the reaction mechanism is complex, and the reaction conditions are variable, so it is often difficult to establish an accurate mechanism model. With the requirements of different downstream product quality and the changes in load and raw materials, many industrial processes work under various conditions.

However, in the sintering process, due to the complex composition of reaction raw materials, difficult to monitor the composition in real time, and few controllable combustion conditions, there is no accurate mechanism model yet.

Fortunately, due to the large-scale application of Programmable logic controllers (PLC) and distributed control system (DCS) systems, it is easy to obtain the process parameters of the sintering process. Through numerous sensors, process parameters such as temperature, pressure and flow can be collected. Therefore, a data soft sensor model can be established with numerous data to predict key parameters that are difficult to be measured in real time, such as the FeO composition of the sinter. At first, linear regression models were widely used to study time series data, such as the autoregressive moving average model (ARMA), autoregressive comprehensive moving average model (ARIMA), etc. [2]. These models generally require strict assumptions. However, in the face of such a nonlinear and multi-coupling process as the industrial process, it is difficult to obtain accurate estimation using linear analysis, and the effect is subject to many restrictions. Therefore, in these nonlinear cases, the performance of traditional linear soft sensing methods, such as principal component analysis (PCA), partial least squares (PLS) and independent component analysis (ICA), is not enough [3].

According to the nonlinear characteristics, some scholars have proposed some Gaussian mixture models and Bayesian models to solve these problems. Some studies have focused on the Bayesian model [4]. Gaussian mixture model and Bayesian method need to determine the super parameter or model structure according to expert knowledge and structure learning. The accuracy of these parameter structures will affect the prediction performance. The support vector machine (SVM) is applied to the prediction model [5]. However, the support vector machine method has a large amount of computation for numerous data, and the effect of the model depends on the selection of key parameters and kernel functions.

In recent years, with the emergence of deep learning, breakthroughs have been made in many fields, such as image recognition, speech recognition and so on. General deep models include auto-encoder, Recurrent Neural Network(RNN), Convolutional Neural Networks(CNN), etc. The deep learning method developed from the artificial neural network fits complex nonlinear systems through multi-layer nonlinear mapping and relies on big data for training. The model result is better than the traditional model. However, in the common field of deep learning, the number of data is more than that of industrial processes, and there are also many labeled data sets, which is convenient for training deep models and model transfer learning.

Auto-encoder (AE) is a group of artificial neural networks. Its output is to reconstruct the data from the input layer. By minimizing the reconstruction error of the data, the characteristics of the data can be learned. AE and its extensions have been used in process monitoring, diagnosis and quality prediction fields [6]. For example, Yuan et al. proposed a variable weighted SAE (Stacked Auto-Encoder) for soft sensor applications to strengthen the relationship between input and output data to be predicted during layered pre-training [7]. All these efforts further help the AE-based approach meet the requirements of different tasks.

For process industry problems, the typical characteristic of data is time dependence. Process industry production is a continuous process. The output of current time is closely related to the input of previous moments. However, the common artificial neural network does not consider time dependence, and the network input is the sampling at the same time. Recently, recurrent neural networks (RNNs) have become very popular for the problem of sequential data learning. RNNs also have many variants, mainly Long Short-Term Memory (LSTM) and Gated Recurrent Unit (GRU) units [8]. Yuan et al. proposed a spatiotemporal attention-based LSTM network for soft sensor modeling [9]. These improved structures can solve the problem of gradient vanishing and the explosion of long time series. However, the RNN network in industry also has the problem of scarce label data. Unlike some internet scenarios, labeled data is relatively easy to obtain. In industry, the labeled data are often obtained by manual test, and the labeling cost is expensive, resulting in the scarcity of labeled data. There are many unmarked data samples. Therefore, in the data-based industrial soft sensing, the quality prediction performance is often troubled by the lack of labeled data samples, and the attention to the unlabeled data is insufficient.

Therefore, in view of the above problems, how to use less labeled samples and more unlabeled samples at the same time has become a hot issue in recent years [10]. We hope to introduce unsupervised learning and semi-supervised learning in industrial processes.

The typical semi-supervised method is to manually label the unlabeled samples by learning the labeled data, and evaluate the improvement of the results of these pseudo labels through strategies [11]. This method is often used in semi-supervised classification problems [12]. Recently, there are some basic semi-supervised modeling methods, usually based on the existing model, using the division of the data set, introducing unlabeled data, and expanding the labeled data set through division [13]. Yao et al. used a corresponding semi-supervised data sequence division scheme to make full use of the information in both labeled and unlabeled data [14]. Sun et al. proposed a new integrated semi-supervised gated stacked auto-encoder for key performance index prediction [15]. Yuan et al. added the prediction error term of the labeled data to the original loss function in the pre-training procedure [16]. However, these methods only play a role in the pre-training process, ignoring the more important fine-tuning phase. In addition, most artificial neural network methods only use the last hidden layer for final output or prediction [17]. For example, Shao et al. proposed a semi-supervised probabilistic hybrid algorithm for extreme learning machines based on variational Bayes expectation maximization algorithm [18].

Most of the above semi-supervised methods use pseudo label method or unsupervised feature extraction method of unlabeled data and do not use the fine-tuning strategy for condition adaptation training. At the same time, the pre-training under the big data of the sintering process has not been reported. In order to solve the above problems, we propose an improved semi-supervised model based on pre-training and fine-tuning strategy. On the basis of the DTFEE framework considering the dynamic time characteristics of the original process, a semi-supervised encoder-decoder with weighted BiLSTM is integrated. The encoder-decoder of stacked weighted BiLSTM makes use of the two-way propagation process of information flow in the forward layer and the backward layer to better solve the long-term dependence, and fully obtain the hidden information of the unlabeled data set in the pre-training and reconstruct it. In this way, the information of unlabeled datasets can be fully mined through pre-training, which greatly improves the performance of the fine-tuning model after migrating to the labeled datasets.

The main contributions of this paper are as follows:

1. To build the encoder-decoder model based on improved weighted BiLSTM and improve the DTFEE framework.
2. Through the pre-training and fine-tuning strategy, the information of unlabeled datasets can be fully extracted and used to improve the effect of labeled datasets, to achieve better semi-supervised learning.
3. The improved performance is verified in the actual sintering process, which has reference value for practical workers. The proposed model combines the process characteristics and makes corresponding adjustments, which are applied and demonstrated in a real iron ore sinter process for FeO prediction with good effect.

The remainder of this paper is organized as follows. In Section 2, the characteristics of the sintering process and quality variables are analyzed. The method and model proposed in this paper are introduced in Section 3. Then, the proposed method is verified by the actual production process data in Section 4. Section 5 summarizes the full text and puts forward new prospects and future work directions.

## 2. Description of the Sintering Process

The sintering process is mixing the iron ore powder of the raw material with limestone, coke powder and pulverized coal after a certain proportion of proportioning, and then producing a series of physical and chemical changes through the sintering machine to form the sinter that meets the requirements of the raw material for blast furnace iron making. The main chemical change is the oxidation-reduction reaction of iron ore. The chemical reaction equation of the sinter is as follows:

$$\begin{array}{c} 3{\mathrm{FeS}}_{2}+8O_{2}={\mathrm{Fe}}_{3}O_{4}+6{\mathrm{SO}}_{2} \end{array}$$

$$\begin{array}{c} {\mathrm{Fe}}_{3}O_{4}+\mathrm{CO}=3\mathrm{FeO}+{\mathrm{CO}}_{2} \end{array}$$

$$\begin{array}{c} 2{\mathrm{Fe}}_{3}O_{4}+3{\mathrm{SiO}}_{2}=3(2\mathrm{FeO}\cdot {\mathrm{SiO}}_{2})+O_{2} \end{array}$$

**Figure 1:**
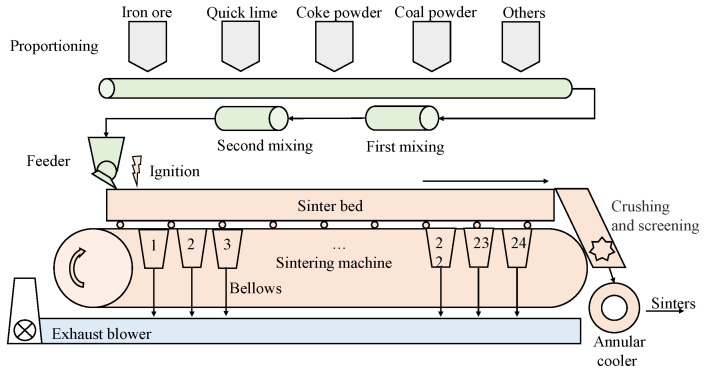
Brief flowchart of sintering process.

The sintering process is shown in Figure 1. The whole sintering process can be divided into the following parts:

1. Proportioning: Raw materials such as iron ore, quicklime, coke powder and pulverized coal are proportioned according to the pre-calculated proportioning ratio and transported to the conveyor belt.
2. Mixing: After the first mixing and second mixing, appropriate moisture is added to make the raw materials mix evenly.
3. Ignition and sintering: The raw materials are laid on the sintering trolley and ignited. With the movement of the trolley, the ignited raw materials are sintered through the lower bellows.
4. Crushing, screening and cooling: After sintering, the sinter is crushed and screened according to the particle size. If the conditions are met, it passes through the annular cooler and enters the silo, ready to be sent to the blast furnace iron-making process.

### 2.1. Description of the Sinter Quality

The sinter produced by the complex process cannot be directly sent to the blast furnace as raw material for iron making. As the main raw material of blast furnace iron making, sinter needs strict quality control. Sinter quality can be divided into physical index and chemical index. The physical indexes are mainly the sinter particle size and drum strength, while the chemical indexes are mainly the mass percentage of main components, such as FeO, total iron content, Cao, SiO_2_, etc., as shown in Table 1. The higher the content of FeO is, the worse the reducibility of the sinter will be. The FeO content will affect the iron-making process of the blast furnace. If the content of FeO is too low, the strength of the sinter will be reduced. Therefore, it is necessary to maintain a suitable and stable FeO content.

### 2.2. Characteristic Analysis

1. Nonlinearity

Complex physical and chemical changes take place in the sintering process. It is difficult to establish the mechanism or empirical model of sinter quality. At present, there are some qualitative studies. Through the research, the linear correlation between sinter quality variables and operating parameters is very low. Therefore, it is difficult to use a simple linear model for fitting predictions.

2. Multiple time delays

**Figure 2:**
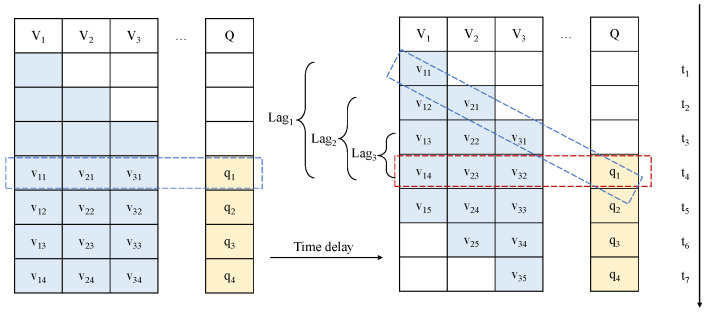
Multiple time delays.

Not only the sintering process, but also almost all process industries have this time delay characteristic. For a batch of raw materials, a period of time will elapse from the beginning of the process to the end of the process. Therefore, different sampling variables correspond to different times. If the process end time is taken as the benchmark, the previously sampled variables need to be corrected for a period of time delay. Moreover, because the working conditions are changing in real time, the time delay of different variables may also be changing. Therefore, it is difficult to modify the time delay in practice, and we use the integrated time series model to learn automatically. The sintering process will last about one hour, so the sampling variables at the same time do not correspond to the same batch of materials, and the variables have the characteristics of time distribution, as shown in Figure 2. For the variables $V=\{v_{1},v_{2},v_{3}\}$, they have multiple time delays:

$$lag_{v1}=t_{4}-t_{1},lag_{v2}=t_{4}-t_{2},lag_{v3}=t_{4}-t_{3}$$

3. Lack of labels

The sinter quality label is obtained by a laboratory test, which is difficult and expensive to obtain. Since it takes a long time for the laboratory to sample and produce the test results, the cost of labeling samples is very high, and it is not possible to label samples intensively. Therefore, the quality label of the sinter is very scarce, which is the key problem of sinter quality prediction. We consider the use of existing tags and numerous unlabeled process data for semi-supervised training.

## 3. Methodology

This section develops the SS-DTFEE framework for FeO forecasting. The encoder-decoder framework is constructed by wBiLSTM cells. According to the characteristics of less industrial data labels, adding pre-training and fine-tuning mechanisms to the model can fully extract process information and improve the ability of potential variables to express process information. In the prediction task, the decoding network is reconstructed, and the potential variables obtained by pre-training are used to fine-tune with a few labeled samples to obtain FeO prediction output. The specific contents of this method are as follows.

### 3.1. Improved LSTM Structure

The recurrent neural network (RNN) is a kind of neural network for processing sequence data. The difference between RNN and an ordinary fully connected neural network is that the input of RNN is a group of sequences $X_{t}=\left(x_{1},x_{2},\ldots ,x_{n}\right)$, and the time correlation of sequence data is learned through repeated RNN cells. However, due to its simple structure and repetition, RNN will lose long-term memory for a long input sequence, making it unable to remember the input information of $x_{1}$. When the input time interval is too long, it is difficult to transmit the early input information to the last time step in the cycle calculation because the gradient disappears.

**Figure 3:**
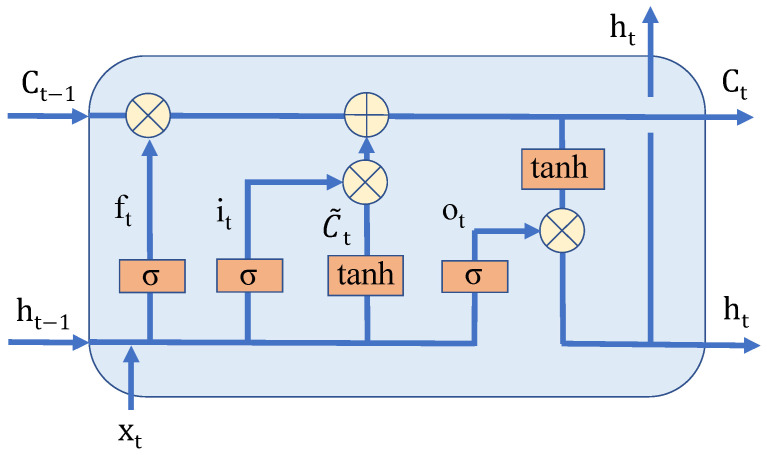
Graph of the LSTM cell [8].

Therefore, on the basis of RNN, a Long Short-Term Memory neural network (LSTM) is proposed to solve the long-term dependence. LSTM consists of cell state $C_{t}$, input gate $i_{t}$, forgetting gate $f_{t}$ and output gate $o_{t}$, as shown in Figure 3. By selectively allowing information to flow through the gate structure, we can achieve better long-term dependence than RNN. The calculation formulas of LSTM are as follows [8]:

(1) $$\begin{array}{cc} f_{t} & =\sigma \left(W_{f}\;\cdot \; [h_{t-1},x_{t}]+b_{f}\right) \end{array}$$

(2) $$\begin{array}{cc} i_{t} & =\sigma \left(W_{i}\;\cdot \; [h_{t-1},x_{t}]+b_{i}\right) \end{array}$$

(3) $$\begin{array}{cc} {\tilde{C}}_{t} & =\tanh\left(W_{c}\;\cdot \; [h_{t-1},x_{t}]+b_{c}\right) \end{array}$$

(4) $$\begin{array}{cc} C_{t} & =f_{t}\times C_{t-1}+i_{t}\times {\tilde{C}}_{t} \end{array}$$

(5) $$\begin{array}{cc} o_{t} & =\sigma \left(W_{o}\;\cdot \; [h_{t-1},x_{t}]+b_{o}\right) \end{array}$$

(6) $$\begin{array}{cc} h_{t} & =o_{t}\times \tanh C_{t} \end{array}$$

**Figure 4:**
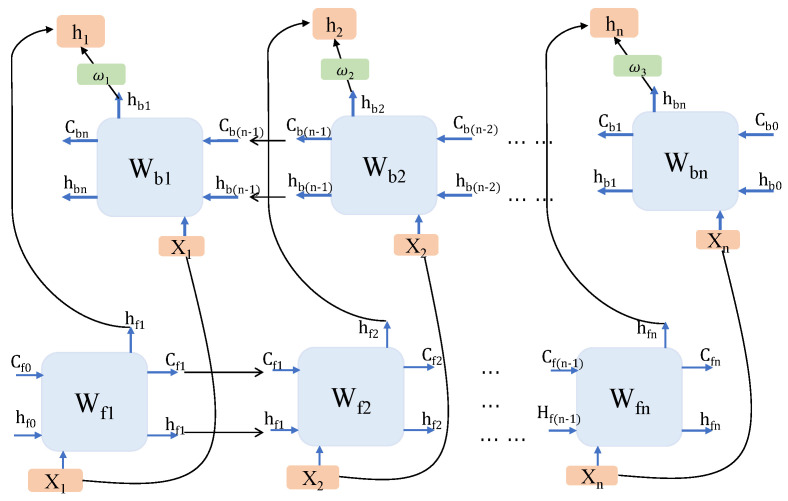
Graph of the weighted BiLSTM.

The weighted BiLSTM cell is a weighted bidirectional LSTM structure. Although LSTM uses gate units to control the forward information flow, the effect of long sequence learning still has room for improvement. BiLSTM uses a two-way structure. The forward layer uses forward propagation $X_{f}=\left(x_{1},x_{2},x_{3},\ldots ,x_{n}\right)$. The input of the backward layer is the reverse order of the input of the forward layer $X_{b}=\left(x_{n},x_{n-1},x_{n-2},\ldots ,x_{1}\right)$. The BiLSTM simply concatenates the forward layer and the backward layer together. In this way, the proportion of information saved in two directions cannot be adjusted, and it is easy to make the network tend in one direction. We design a weighted unit to adjust the proportion of bidirectional information fusion through the parameter ω. The state vector of weighted BiLSTM is calculated by this formula. $h_{t}=h_{f}+\omega h_{b}$ This makes the model have the more flexible learning ability of two-way timing information. The weighted unit can obtain the information at the beginning of the sequence better. The output information of the two layers is weighted and spliced to obtain the output of weighted BiLSTM $h_{t}$, as shown in Figure 4.

### 3.2. Encoder-Decoder Model

The encoder-decoder framework was usually applied in the field of machine translation. The problem of machine translation is to convert a language sequence into another language sequence. In this paper, the technology is extended to other fields, such as the industrial field. The input sequence can be industrial timing data, image, audio, etc., and the output sequence is also industrial timing data, image, audio, etc., which can solve the problems of different data types. This large class of problems can also be called sequence to sequence problems. The framework used in this paper takes the input as industrial time series data and the output as industrial time series data. The input sequence of the encoder part is industrial time series data. Due to the time correlation, the most widely used encoder is the recurrent neural network. RNN is a basic model. When training, we encounter the problem of gradient explosion or gradient vanishing, resulting in the inability to train. Therefore, we use the improved LSTM to represent the input sequence X∈X. The input sequence is encoded into a hidden state vector h∈F by the encoder. In the decoder part, hidden state vectors generated by the encoder are used as inputs to decode the target industrial time series data. Like encoder, this framework adopts LSTM to build the decoder model, and the model output $\hat{X}\in X$ is used to reconstruct the input sequence *X*.

**Figure 5:**
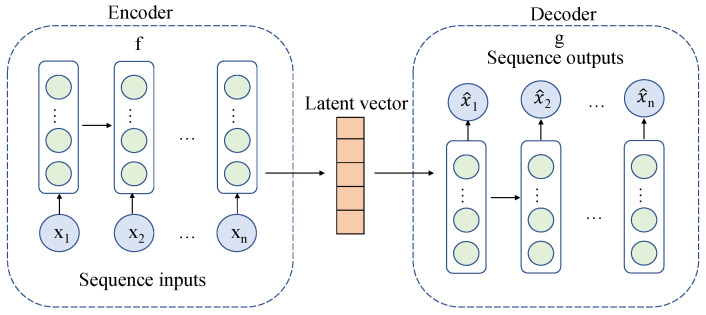
Graph of the encoder-decoder model.

The encoder-decoder model wants the reconstruction error of X to be as small as possible, that is, the decoded $\hat{X}$ should be as close to *X* as possible. The loss function is the mean square deviation of *X* and $\hat{X}$. The optimization objective is to find f:X→F and g:F→X of encoder and decoder under the condition of minimizing loss function. The model structure is shown in Figure 5.

(7) $$\begin{array}{cc} {\hat{x}}_{i} & =g(f\left(x_{i}\right)) \end{array}$$

(8) $$\begin{array}{cc} L(x,g(f(x)) & ={∥x-g\left(f\left(x\right)\right)∥}^{2} \end{array}$$

(9) $$\begin{array}{cc} f,g & =\underset{f,g}{\arg\min}L\left(x,g\left(f\left(x\right)\right)\right) \end{array}$$

### 3.3. Pre-Training and Fine-Tuning

The pre-training method is to pre-train the network with dataset $D_{a}$ and task A, learn the network parameters on dataset *A*, and then save the network structure and parameters for future use. When facing new data $D_{b}$ and task B, the same network structure is adopted. When initializing the network parameters, the parameters learned by data $D_{a}$ can be loaded, and the other parameters are initialized randomly. Then, the network is trained with the training data $D_{b}$ of task B. Facing different task goals, we can choose the strategy of parameter learning. It is called frozen when the loaded parameters remain unchanged. When the loaded parameters change continuously with the training of task B, it is called fine-tuning, that is, adjusting the parameters to make them more suitable for the current task B. In the process of modifying the model through fine-tuning, we usually use a smaller learning rate than the general training model.

Methods for fine-tuning the model:

1. Feature extraction. Under this strategy, the whole pre-training model is used as a feature extractor. The output layer of the pre-training model is removed, and the structure and parameters of the remaining part are retained. It is used as a fixed feature extractor without training its parameters. New data sets are input into it to obtain the extracted features.
2. Keeping the structure and parameters of the pre-training model. This strategy retains the structure of the pre-training model, but first randomizes all the weights, and then trains according to its own data set.
3. Training specific layers and freezing other layers. This strategy uses a pre-training model and fine-tuning it. Keep the weights of some layers at the top of the model unchanged, set it as untrainable and retrain the following layers to obtain new weights. Generally speaking, if the data set is small and similar to the original pre-training set, the number of frozen layers can be more and the number of retraining layers can be less. This fine-tuning strategy is adopted in our models.
  The formulas of the pre-training model and fine-tuning model are as follows. $N_{l}$ is the number of samples of labeled dataset $D_{l}$, and $N_{u}$ is the number of samples of unlabeled dataset $D_{u}$.

Pre-training:

(10) $$\begin{array}{cc} {\hat{x}}_{i} & =g_{1}\left(f_{1}\left(x_{i}\right)\right) \end{array}$$

(11) $$\begin{array}{cc} L(x,\hat{x}) & =\frac{1}{N_{l}+N_{u}}\sum_{}^{N_{l}+N_{u}}{∥x-\hat{x}∥}^{2} \end{array}$$

(12) $$\begin{array}{cc} f_{1},g_{1} & =\underset{f_{1},g_{1}}{\arg\min}L\left(x,g_{1}\left(f_{1}\left(x\right)\right)\right) \end{array}$$

Fine-tuning:

(13) $$\begin{array}{cc} {\hat{y}}_{i} & =g_{2}\left(f_{2}\left(x_{i}\right)\right) \end{array}$$

(14) $$\begin{array}{cc} L(y,\hat{y}) & =\frac{1}{N_{l}}\sum_{}^{N_{l}}{∥y-\hat{y}∥}^{2} \end{array}$$

(15) $$\begin{array}{cc} f_{2},g_{2} & =\underset{f_{2},g_{2}}{\arg\min}L\left(y,g_{2}\left(f_{2}\left(x\right)\right)\right) \end{array}$$

### 3.4. Semi-Supervised Dynamic Time Feature Expanding and Extracting Framework

The dynamic time feature expanding and extracting framework (DTFEE) for the sintering process includes feature selection, time delay, time difference, and time serialization [19]. In the feature selection part, the correlation coefficient and maximum mutual information coefficient are used for the reduction of variables dimensions. Lightgbm, XGBoost and random forest can be used as integrated feature extractors. The difference processing can eliminate the time lag and nonstationarity.

Because the working conditions are changing, the time delay of different features is also changing. For this reason, the traditional static time delay processing is not effective. This paper uses the integrated weighted BiLSTM encoder-decoder model to embed the learning time delay into the sequence model.

**Figure 6:**
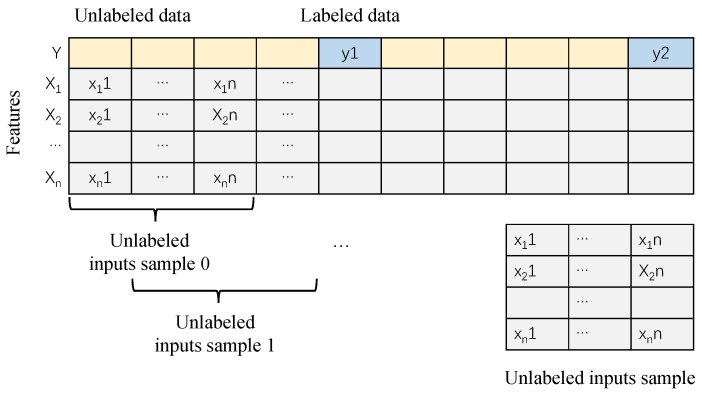
Expansion and serialization of time series data.

Time serialization generates sequence input and constructs training set through a sliding window. The composition of the data set is determined by the length of the input sequence and divided into unlabeled dataset $D_{u}$ and labeled dataset $D_{l}$. As shown in Figure 6. This can effectively extract the sequence characteristics of raw data.

This paper builds an end-to-end model through dynamic temporal feature extraction, which enables the model to automatically learn and adjust latency. Compared with the manual calculation of static and dynamic time delay, it has better practicability and is convenient for online operation.

**Figure 7:**
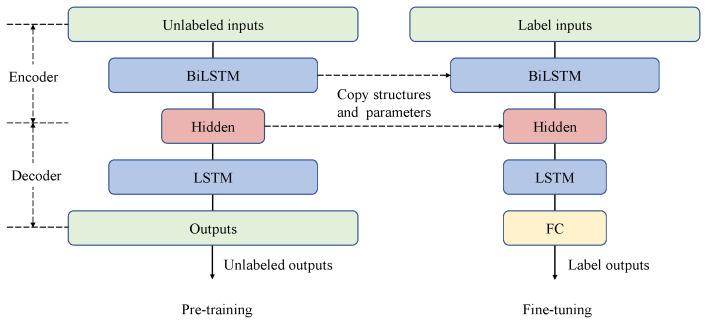
Brief flowchart of semi-supervised encoder-decoder model with pre-training and fine-tuning methods.

The self-supervised encoder is constructed by our weighted BiLSTM unit, and the decoder is constructed by LSTM. The semi-supervised encoder is constructed by our weighted BiLSTM unit, and the decoder is constructed by LSTM and the full connection layer. The encoder-decoder model, which adds the pre-training and fine-tuning mechanism, makes full use of the unlabeled process information for self-learning. The model uses the learned process of hidden information to improve the prediction effect of labeled data, and constructs a semi-supervised learning framework, as shown in Figure 7.

**Figure 8:**
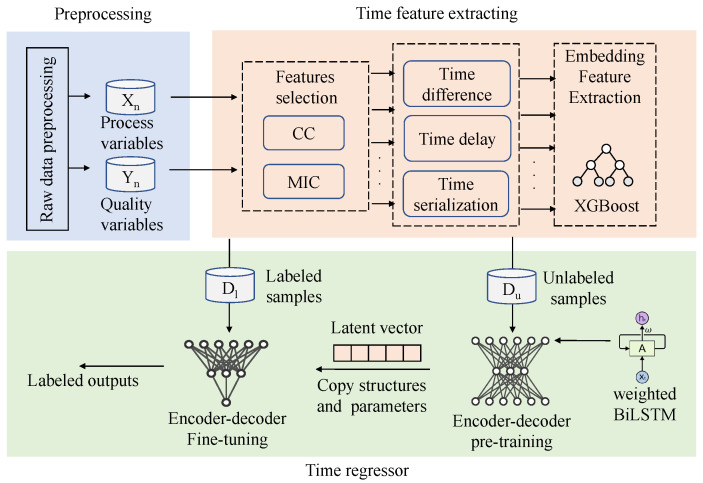
Brief flowchart of semi-supervised dynamic time features expanding and extraction prediction framework (SS-DTFEE).

The flowchart of semi-supervised dynamic time features expanding and extraction prediction framework (SS-DTFEE) is shown in Figure 8.

## 4. Illustration and Discussion

### 4.1. Dataset Introduction

**Figure 9:**
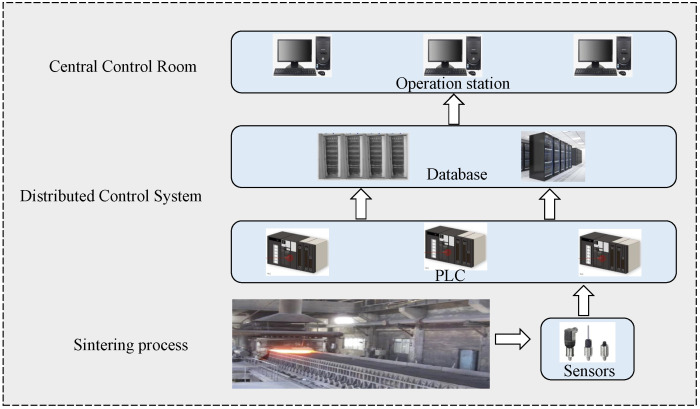
Brief flowchart of DCS.

The dataset used in this experiment was collected in a sintering plant of an iron and steel enterprise. The plant has a 360 m^2^ belt sintering machine, which is 90 m long and 4 m wide. The data is collected from the distributed control system (DCS) of the sintering plant, as shown in Figure 9. Programmable logic controllers (PLC) with different functions collect data from the sensors of the sintering machine and collect them into the DCS in the central control room. PLC can realize some basic automation functions, such as material batching control, ignition control, machine speed control, etc. The DCS measurement system collects a set of raw data every second and stores it in the database. The data set used for the experiment was collected in two months, with about 101,400 records and 432 variables, including 320 groups of labeled data and the rest of unlabeled data. Labeled data is obtained by manual sampling and testing four times a day. Due to the high cost and long test cycle of FeO, the data with labels are relatively rare and precious.

Then, the original data is preprocessed. As described above, FeO as label data has a long sampling period, resulting in a few records, only about 320. According to Figure 6, we conduct time expanding on all variables to fully mine the dynamic characteristics of unlabeled data. Combined with time expansion, time difference, and time serialization, 300 groups of original data are made. Some input variables are shown in Table 2.

### 4.2. Data Preprocess

Before the experiment, the 3−σ criterion is used to remove the outliers from the original data set.

Then, to train the later neural network, the 0–1 min-max normalization preprocessing is performed and scaled to the [0,1] interval.

**Figure 10:**
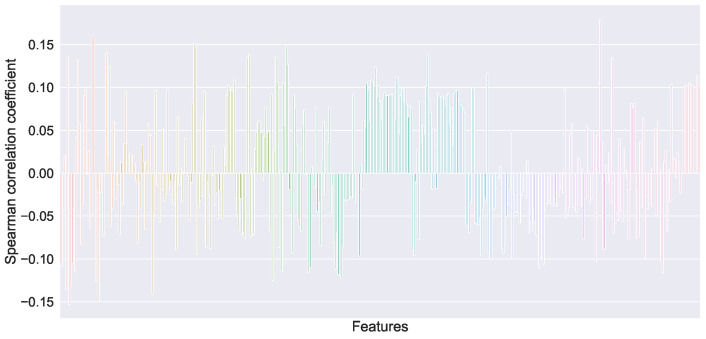
Spearman correlation coefficient between raw variables and FeO.

**Figure 11:**
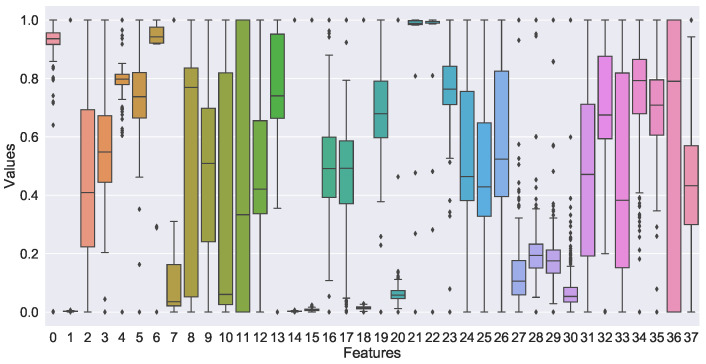
The box diagram of the selected variables.

The initial features of the original data have 431 dimensions, including many redundant and invalid features. To train the model accurately and quickly, we need to select features. The feature selection method has a correlation coefficient and maximum mutual information coefficient. Lightgbm, xgboost and random forest can be used as integrated feature extractors [20]. FeO correlation is shown in Figure 10. A total of 36 variables with an FeO correlation coefficient greater than 0.1 were selected, and the input dimension of the training set *X* was 36. The box diagram of the selected variables is shown in Figure 11.

Next, time delay and difference processing are performed, the sequence input is generated by sequencing, and the training set is constructed. This can effectively extract the dynamic time characteristics of data.

### 4.3. Experimental Settings

After data preprocessing, unlabeled data is used as the pre-training input $D_{u}$ and the pre-training target, and the pre-training input and the output sequence length is 20.

Labeled data is used as the fine-tuning training set $D_{l}$, the input sequence length is 20, and the output sequence length is 1. The data set is divided into training set 80%, verification set 10% and test set 10%.

In our experiment, all the comparison models were implemented using the TensorFlow GPU framework (Google Brain, Mountain View, CA, USA) in Python. The test platform is a computer with i9-10900 CPU, 32 g RAM and RTX3060 GPU.

In this experiment, the accuracy of the prediction model is evaluated by the Mean Square Error (MSE), the Mean Absolute Error (MAE) and Hit Rate(HR), where $y_{i}$,${\hat{y}}_{i}$ are the real value and prediction, respectively, and *n* is the testing samples number.

(16) $$\begin{array}{cc} MSE & =\frac{1}{n}\sum_{i=1}^{n}{\left(y_{i}-{\hat{y}}_{i}\right)}^{2} \end{array}$$

(17) $$\begin{array}{cc} MAE & =\frac{1}{n}\sum_{i=1}^{n}\left|y_{i}-{\hat{y}}_{i}\right| \end{array}$$

(18) $$\begin{array}{c} \begin{array}{c} H_{i}=\left\{\begin{array}{cc} 1, & \left|y_{i}-{\hat{y}}_{i}\right|/y_{i}<=3\%\\ 0, & \left|y_{i}-{\hat{y}}_{i}\right|/y_{i}>3\% \end{array}\right. \end{array} \end{array}$$

(19) $$\begin{array}{cc} HR & =\frac{1}{n}\sum_{i=1}^{n}H_{i} \end{array}$$

As the benchmark experiment for comparison, the dataset is the labeled dataset $D_{l}$ without unlabeled data. LSTM neural network and weighted BiLSTM neural network are used for training and prediction.

Recently, the popular method for processing time series data is the LSTM model, which is the improved RNN model. To be fair, all methods use the same parameter settings. LSTM neural network consists of 100 cells. After LSTM neural network, the output is connected to the full connection layer for prediction. BiLSTM neural network is set to 50 cells and 2 layers. After bidirectional LSTM, the output is connected to the full connection layer for training. The optimizer selects Adam optimizer, and the learning rate is 0.0001. Epoch is 100, and batch size is 8.

In contrast, the improved SS-DTFEE model with a pre-training fine-tuning structure is proposed in this paper. Based on different pre-training structures, it is divided into two types: encoder and decoder with single-layer LSTM structure, an encoder with weighted BiLSTM structure and decoder with LSTM structure. Based on the pre-training and fine-tuning policy, the connected decoder is the LSTM connected with a full connection layer. As a comparison experiment, the first strategy is not to fine-tune. The pre-training weights are only used as the network initialization parameters, and the weights are updated during the network training. The second strategy is to fine-tune and freeze the weights of the encoder part. Only the parameters of the decoder are updated during training.

PF abbreviation indicates that the unit uses a pre-training and fine-tuning strategy. There are four types of encoder-decoder structures proposed above, namely:

- Encoder-decoder-LSTM+LSTM
- Encoder-decoder-BiLSTM+LSTM
- Encoder-decoder-LSTM+LSTM-PF
- Encoder-decoder-BiLSTM+LSTM-PF(SS-DTFEE)

### 4.4. Results Comparison and Analysis

**Figure 12:**
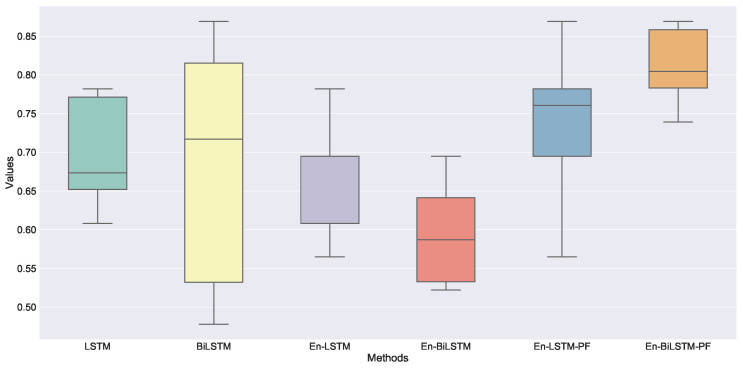
Boxplots of hit rate metrics for each method.

As well as LSTM and BiLSTM, a total of six models were tested. Each model is trained 10 times, and the average value of the evaluation index is taken as the record, as shown in Table 3. We draw the HR of ten experiments of six models into a box diagram, as shown in Figure 12.

By comparison, the SS-DTFEE model proposed by us has achieved good results. The basic LSTM model and BiLSTM model only use the label data set $D_{l}$. Because the data set is small, it is difficult for the model to learn enough information, so the prediction accuracy of the model is insufficient.

**Figure 13:**
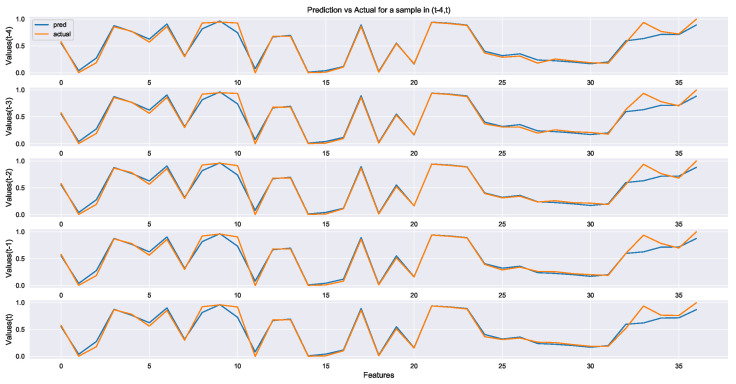
Five-time-step reconstruction of each feature in the pre-training phase.

As the encoder-decoder model for comparison, the two groups only use the pretrained strategy, and the pre-training data set is unlabeled data $D_{u}$. Through the self-learning of unlabeled data, the hidden information of the process is obtained from numerous unlabeled data, and the information is transferred to the prediction network with label data. The length of the decoder output sequence is 20, that is, the length of the reconstructed time sequence is 20. The length of the last five-time step sequence is shown in Figure 13. Figure 13 illustrates that the output of the encoder-decoder model in this experiment reconstructs the input sequence with a good effect. Then, the latent vector of the encoder-decoder model extracts the characteristics of the sintering process data, which can be used for the pre-training and fine-tuning model. However, for models that do not use the fine-tuning strategy, more parameters need to be trained as the model architecture becomes more complex. Furthermore, the number of labeled datasets is too small to train all parameters. Therefore, the output effect of the model is poor, and the output stability is worse than that of LSTM and BiLSTM without an encoder-decoder structure.

**Figure 14:**
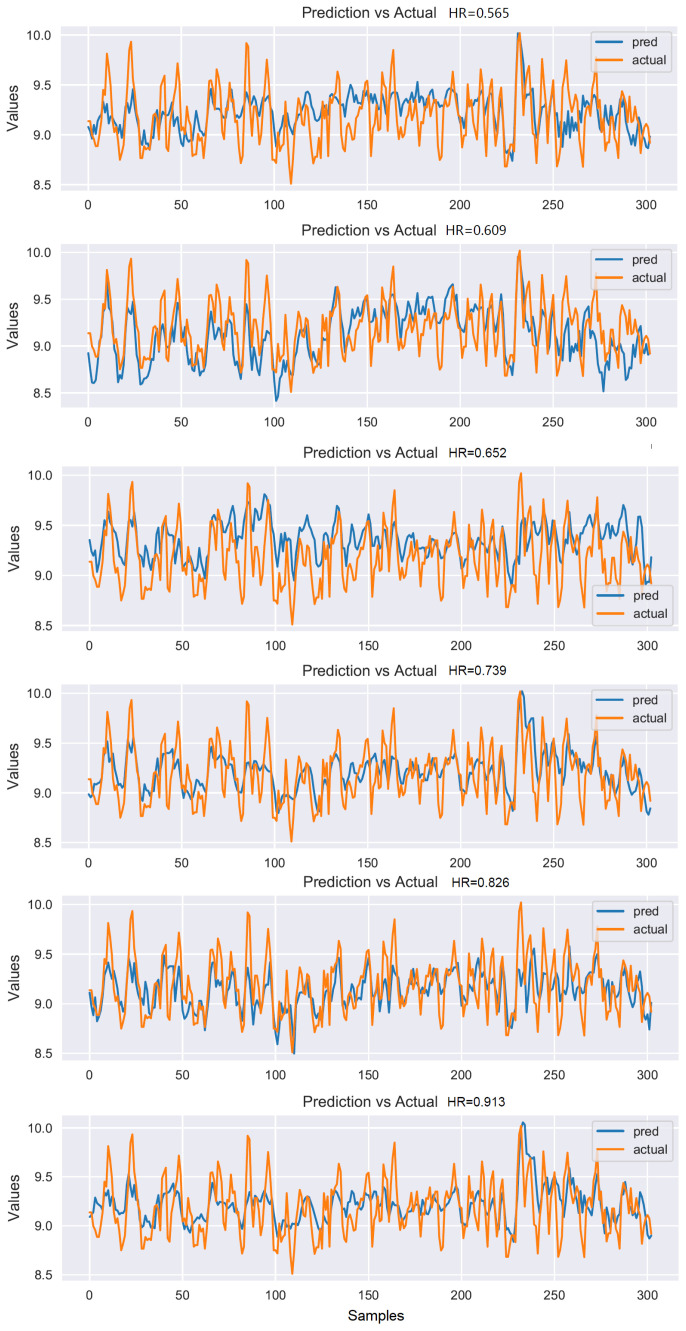
Single experiment results of each method.

By contrast, the two groups of models using the fine-tuning strategy have achieved obvious improvement. The prediction of hit rate increased from 0.669 to 0.739 and 0.593 to 0.813, respectively. The unlabeled datasets are trained by using the pre-training strategy, and the hidden information of the process is obtained. By migrating the encoder model and freezing the weights, the amount of parameters to be trained is greatly reduced, and then training with labeled data can achieve better results. Moreover, because the BiLSTM model is more complex and has more parameters than the LSTM model, the prediction accuracy of the BiLSTM model is improved more significantly. Additionally, the fine-tuned BiLSTM model proposed by us exerts a good bidirectional learning ability of sequences and achieves the best results. The demerits of our method are that we need to train two models, which takes more time than a single model and the fine-tuning model data needs to be similar to the pre-training model data. If the working condition changes greatly, the training effect of the model will be affected. Ten repeated experiments were carried out for each method, and the average of the evaluation indexes was taken as the result and listed in Table 3. Among the ten repeated experiments of each method, one experiment result is selected, as shown in Figure 14. The Figure 14 from top to bottom are method LSTM, method BiLSTM, method Encoder-decoder-LSTM+LSTM, method Encoder-decoder-BiLSTM+LSTM, method Encoder-decoder-LSTM+LSTM-PF and method Encoder-decoder-BiLSTM+LSTM-PF(Ours), respectively, as shown in Table 3.

## 5. Conclusions

In this paper, a novel encoder-decoder framework based on semi-supervised dynamic time features expanding and extraction is proposed to predict FeO and other parameters in the iron ore sintering process. The framework can effectively use numerous unlabeled data to obtain industrial process information. The encoder-decoder model is built by our proposed weighted BiLSTM, which can better learn the bidirectional time series hidden information. The unlabeled data is based on the pre-training strategy to obtain the network structures and weights, which contains the process hidden layer information. Under the fine-tuning strategy, the hidden layer information obtained from the pre-training is trained together with the labeled data to obtain a better prediction effect than supervised learning. The proposed method is applied in the actual sintering process to estimate the FeO content, which shows a significant improvement in the prediction accuracy and stability, compared to traditional supervised methods. In the future, the framework of the model can be further improved in accuracy, and it can be applied to other process industries, not only sintering processes.

## Figures and Tables

**Table 1:** Table of some sinter quality variables.

No.	Quality Variables	Descriptions
1	FeO	Mass percentage of FeO in sinter
2	TFe	Mass percentage of total iron content of sinter
3	CaO	Mass percentage of CaO in sinter
4	MgO	Mass percentage of MgO in sinter
5	Tumbler index	Tumbler strength of Sinter
6	Screening index	Screening index of Sinter

**Table 2:** Part of the collected original variables.

No.	Variables	Unit
1	Sinter Thickness	mm
2	Ignition Temperature	°C
3	Bellows Negative Pressure	kPa
4	Bellows Temperature	°C
5	Belt Mass Flow	t/h
6	Water addition	m^3^/h
7	FeO	%

**Table 3:** Evaluation of method predictions effect.

Methods	MSE	MAE	HR ^1^
LSTM	0.063	0.207	0.695
BiLSTM	0.061	0.209	0.691
Encoder-decoder-LSTM+LSTM	0.061	0.201	0.669
Encoder-decoder-BiLSTM+LSTM	0.071	0.211	0.593
Encoder-decoder-LSTM+LSTM-PF	0.059	0.203	0.739
**Encoder-decoder-BiLSTM** **+LSTM-PF(Ours)**	**0.048**	**0.182**	**0.813**

^1^ Hit rate 3%.

## Data Availability

Not applicable.

## Abbreviations and Nomenclature

### Abbreviations

The following abbreviations are used in this manuscript:

AE	Auto-Enconder
ANN	Artificial Neural Network
ARIMA	Autoregressive Integrated Moving Average model
ARMA	Autoregressive Moving Average
BiLSTM	Bidirectional Long Short-Term Memory
CC	Correlation coefficient
CNN	Convolutional Neural Network
DCS	Distributed Control System
DTFEE	Dynamic Time Features Expanding and Extraction framework
GRU	Gated Recurrent Unit
HR	Hit Rate
ICA	Independent Component Analysis
LSTM	Long Short-Term Memory
MAE	Mean Absolute Error
MSE	Mean Square Error
PCA	Principal Component Analysis
PF	Pre-training and Fine-tuning
PLC	Programmable logic controller
PLS	Partial Least Squares

RNN	Recurrent Neural Network
SAE	Stacked Auto-Encoder
SS	Semi-supervised
SS-DTFEE	Dynamic Time Features Expanding and Extraction framework
SVM	Support Vector Machine

### Nomenclature

*V*	Process variables
*Q*	Quality variables
lag	Time lag of the variable
$C_{t}$	Cell state of LSTM
$i_{t}$	Input gate of LSTM
$f_{t}$	Forgetting gate of LSTM
$o_{t}$	Output gate of LSTM
$y_{i}$	Real output
${\hat{y}}_{i}$	Predicted output
*n*	The test samples number
$D_{u}$	Unlabeled dataset
$D_{l}$	Labeled dataset
$N_{u}$	Number of samples of unlabeled dataset
$N_{l}$	Number of samples of labeled dataset
*X*	Input sequence
$\hat{X}$	Output sequence
X	The set of sequence
*h*	Hidden state vector
F	The set of hidden state
*f*	Encoder function
*g*	Decoder function
